# The Clinical Utilization of Circulating Cell Free DNA (CCFDNA) in Blood of Cancer Patients

**DOI:** 10.3390/ijms140918925

**Published:** 2013-09-13

**Authors:** Yahya I. Elshimali, Husseina Khaddour, Marianna Sarkissyan, Yanyuan Wu, Jaydutt V. Vadgama

**Affiliations:** 1 Division of Cancer Research and Training, Department of Internal Medicine, Charles Drew University of Medicine and Science, 1720 East 120th Street, Los Angeles, CA 90059, USA; E-Mails: mariannasarkissyan@cdrewu.edu (M.S.); yanyuanwu@cdrewu.edu (Y.W.); jayvadgama@cdrewu.edu (J.V.V.); 2 Laboratory Diagnostic Medicine, Faculty of Pharmacy, Mazzeh (17th April Street), Damascus University, Damascus, Syria; E-Mail: husseina.khaddour@hotmail.com; 3 David Geffen School of Medicine at UCLA, UCLA’s Jonsson Comprehensive Cancer Center, 8-684 Factor Building, Box 951781, Los Angeles, CA 90095-1781, USA

**Keywords:** circulating cell free DNA, neoplasms, early diagnosis, follow up on cancer treatment, prognosis

## Abstract

Qualitative and quantitative testing of circulating cell free DNA (CCFDNA) can be applied for the management of malignant and benign neoplasms. Detecting circulating DNA in cancer patients may help develop a DNA profile for early stage diagnosis in malignancies. The technical issues of obtaining, using, and analyzing CCFDNA from blood will be discussed.

## 1. Introduction

New discoveries in the biomedical field have remarkably enhanced our understanding of the pathology and etiology of disease, particularly originating from the genetic and molecular world. The use of DNA as a biomarker in clinical medicine for early diagnosis, prognosis and monitoring of therapy has been a significant advancement in the field. Whether the DNA is present in normal locations such as the nucleus and mitochondria or circulating free in the blood and body fluids, it can be utilized as a valuable biomarker. Circulating DNA as a biomarker is easily accessible, reliable, and reproducible. In addition, use of DNA assays for clinical medicine can be significantly sensitive and specific if cancer-specific DNA alterations are tested instead of elevation of circulating DNA concentration [1–8]. Detecting somatic mutations from plasma DNA in advanced cancer patients may be potentially preferable when repeated tumor biopsies are not feasible and genomic analysis of archival tumor is deemed insufficient [9].

Circulating cell free DNA (CCFDNA) molecules were first identified in 1948. Subsequent investigations revealed CCFDNA to be present in higher levels among patients with autoimmune diseases and cancer as compared with healthy individuals [5–9]. Additional cell free DNA species, such as cell-free mitochondrial DNA (mtDNA) are also under evaluation for clinical relevance [5]. Thus far, mtDNA is has been shown to be more sensitive to oxidative damage than nuclear DNA [10]. This observation has opened up exciting possibilities for non-invasive diagnostic evaluation and follow-up methods for cancer and other diseases that can be performed with high accuracy and at a reasonable cost.

The overarching goal of utilizing CCFDNAs as biomarkers is to optimize medical practice, advance personalized medicine, and improve the quality of life [11–18]. However, there is still a challenge to authenticate the actual clinical validity of various CCFDNA alterations as potential cancer biomarkers in practice for individual tumor types. Specifically, it is necessary to perform a methodical harmonization of assessing CCFDNAs in terms of sample collection, processing, and analysis. In addition, the clinical significance for the use of CCFDNAs needs to be further verified within the context of well-designed prospective studies with sufficient power and sample size [10,19,20].

## 2. Free DNA in Healthy-Individual’s Blood *vs.* Non-Healthy Individual’s Blood

Extracellular nucleic acids are found in human blood and cell culture medium as cell-free or bound to the cell-surface. The cell-free and cell-surface-bound extracellular nucleic acid are naturally forming complexes with proteins or membrane-bound particles. Gene-target and whole-genome studies reveal significant differences in gene representation between extracellular DNA and genome DNA [21]. The mean quantity of plasma circulating DNA in normal subject is varied from less than 10 ng/mL to more than 1500 ng/mL.

To date, the majority of the gene sequences of CCFDNA reported in the literature associated with disease (e.g., *p53*, the *Ras* family, *beta-globin*, or *beta-actin*) are not part of circulating DNA in healthy individuals. Most of the plasma DNA of normal individuals belongs to the Alu repeat family. The Alu sequences are about 300 base pairs long and are therefore classified as short interspersed elements (SINEs) among the class of repetitive DNA elements [1,2,7]. Determining the source and function of circulating DNA is essential and can be facilitated by new sequencing techniques.

## 3. The Source of DNA in Circulation

In healthy individuals, the concentration of circulating DNA is low, since most non-living cells are removed efficiently from circulation by phagocytes. Published studies to date are somewhat unclear in that there is no clear indication whether the serum or the plasma is a better source for circulating DNA to be tested. In addition, studies to date have not yet made it clear whether the method of analysis or the clinical presentation of the patient in terms of cancer type, tumor location, or tumor stage influence this phenomenon. The methodology of identifying the DNA is primarily the main source of concern. For example, during serum separation, lysis of peripheral blood lymphocytes may cause an artificial increase in DNA integrity [22–25].

Increases in serum DNA were reportedly observed with overnight clotting after blood draw but not at 8 h. Hence, it is preferable to process the specimen within 6 h after blood drawing [23]. Apoptosis is confirmed as one of the major sources of DNA in the plasma or serum. Additional minor source include cell lysis by the necrotic pathway, spontaneous release of newly synthesized nucleic acids, break down of blood cells, break down of any pathogens such as bacteria or viruses, and leukocyte surface DNA. Nucleic acids may also enter cells and exhibit a biological activity in the recipient cells [2,26,27]. Molecular weight of circulating DNA may indicate its source. For example, apoptosis has been found to produce fragments of ~180 bp, whereas necrosis results in higher molecular weight fragments. When double stranded circulating DNA in plasma and serum is separated by gel electrophoresis, the fragments tend to form a ladder rather than a smear. The ladder fragments are mainly 180–1000 bp in size and so are likely to be formed by apoptosis. DNA released by necrosis is incompletely and non-specifically digested and thus smears on electrophoretic separation due to its fragment sizes of about 10,000 bp [28]. Tumor necrosis occurs either from cellular overgrowth or in response to therapy. The cells release DNA due to alteration of their membrane permeability whether they are at their primary location or circulating in the peripheral blood. The active release of DNA from cells might also be expected *in vitro* and through Line-1 (L1) retrotransposon. Presence of CCFDNA is also being assessed in other sources from the boding including urine, synovial fluids, saliva and sputum for cancer diagnosis. Urine may be better source for CCFDNA than plasma or serum because of the inhibitory/digestive factors found in serum/plasma [28–32].

## 4. Preanalytical Considerations

Techniques used for CCFDNA analysis are one of the major obstacles in translating CCFDNA analysis to clinical practice. No standard operating procedure currently exists despite several ongoing clinical studies on CCFDNA analysis. Preanalytical parameters potentially affecting CCFDNA concentration and fragmentation are present at every step from blood draw to the storage of DNA containing sample [33].

## 5. Obtaining DNA from Circulation

The quantity of free-circulating DNA in plasma, serum, and other body fluids is usually low and its isolation is still a challenge especially to determine the origin of the circulating nucleic acids.

In some forms of CCFDNA, procedural isolation can be better achieved. For example in the cell-surface bound DNA, the interactions are so weak that the extracellular cell-surface-bound DNA can be easily eluted with EDTA solution. Additional strategies including eluting the more tightly bound DNA with mild trypsin treatment of the cells together with the polypeptide binding nucleic acids. There is no correlation found between the ages of the patients and the concentrations of free or cell-surface-bound circulating DNA. However, studies have identified that the total mean concentration of circulating cell-surface-bound DNA in blood was higher in healthy men (1030 ng/mL of blood) than in healthy women (430 ng/mL) [7,15]. The following methods may be used for obtaining circulating nucleic acids for clinical analysis.

### 5.1. QIAamp Method and Modified QAIamp Protocol

The QIAamp system is designed to purify genomic, mitochondrial, and bacterial DNA, total cellular RNA, or viral nucleic acids from a wide range of clinical samples for downstream amplification and blotting applications. QIAamp Kits simplify isolation of nucleic acids with fast spin-column or 96-well-plate procedures. No phenol-chloroform extraction is required. Nucleic acids bind specifically to the QIAamp silica-gel membrane while contaminants pass through. PCR inhibitors such as divalent cations and proteins are completely removed in two efficient wash steps, leaving pure nucleic acid to be eluted in either water or a buffer provided with the kit [34,35].

### 5.2. Triton/Heat/Phenol Protocol (THP) for CFDNA Purification

This method has good-quality products. The blood samples should be kept at/or below room temperature (18–22 degrees C) for no more than 2 h before plasma separation by double-spin. Due to the higher efficiency, low-cost and good-quality products, this method is preferred in many circumstances for extraction of DNA. Furthermore, the modified phenol-chloroform (MPC) technique can extract more plasma cell free DNA than the Qiagen kit method [35–37].

### 5.3. Blunt-End Ligation-Mediated Whole Genome Amplification (BL-WGA)

This is a single-tube reaction. Purified double-stranded DNA is blunted with T4 DNA polymerase, self-ligated or cross-ligated with T4 DNA ligase, and amplified via random primer-initiated multiple displacement amplification. BL-WGA improves sensitivity for detection of circulating tumor-specific biomarkers from bodily fluids or for recovery of nucleic acids from sub-optimally stored specimens [16].

### 5.4. The NucleoSpin Method

This is a very rapid method, resulting in a high purity DNA yield. The NucleoSpin method may use for the retrieval of small DNA fragments [38].

## 6. Clearance of DNA from Circulation

The circulating DNA in plasma is protein-bound (nucleosomal) DNA and circulating DNA has a short half-life (10 to 15 min) which is removed mainly by the liver. Accumulation of DNA in the circulation can result from an excessive release of DNA caused by massive cell death, inefficient removal of the dead cells, or a combination of both [22]. It should be noted that although patients requiring renal support have higher values of circulating DNA, the renal elimination is not the main mechanism of CCFDNA clearance. The plasma levels of CCFDNA do not seem to be dramatically altered in chronic kidney disease, peritoneal dialysis or hemodialysis [39].

## 7. Detection Methods for CCFDNA

At the DNA level the detection of point mutations, microsatellite alterations, chromosomal alterations (inversion and deletion) as well as hypermethylation of promoter sequences are possible [40]. Tumor-derived CCFDNA exhibits a specific profile based on DNA size and significantly higher DNA fragmentation [41].

The following methods have been used for detecting CCFDNA:

(1) Modified semi-nested or nested methylation-specific PCR (MSP) for hypermethylated genes that reveals 96% sensitivity and 88% specificity [42–45].
(2) Quantitative, multiplex PCR for circulating nuclear or mitochondrial DNA in serum and/or plasma [46].
(3) Quantification of circulating DNA by real-time quantitative PCR or immunological methods such as ELISA [22].
(4) DNA Methylation specific PCR (MSP) (qMS-PCR) [14,47,48].
(5) Direct SYBR (R) Gold assay is a very accurate and simple technique for measuring CCFDNA in biological fluids without prior DNA extraction and amplification. The assay is not affected by exposure of whole blood or serum to room temperature for 4 or 24 h, respectively [49].
(6) Utilization of LOH of microsatellite biomarkers followed by post-PCR product analysis using capillary array electrophoresis [50].
(7) Cloning and sequencing of free circulating DNA which was successful in giving valuable information about the origin and function of the nucleic acid molecules [1,51,52].
(8) Quantitative methylation analysis of minute DNA amounts after whole Bisulfitome Amplification (qMAMBA) [53,54].
(9) Single tube extraction and processing technique dubbed “methylation on beads” that allows for DNA extraction and bisulfite conversion for up to 2 mL of plasma or serum. In comparison to traditional techniques including phenol chloroform and alcohol extraction, methylation on beads yields a 1.5- to 5-fold improvement in extraction efficiency. The technique results in far less carryover of PCR inhibitors yielding analytical sensitivity improvements of over 25-fold [55].
(10) Quantification PCR of DNA in plasma/serum samples by using PicoGreen assay [56].
(11) Matrix-assisted laser desorption/ionization time of flight (MALDI-ToF) mass spectrometry that is combined with base extension after PCR allows cell free DNA detection with single base specificity and single DNA molecule sensitivity. DNA is first amplified by PCR, then linear amplification with base extension reaction (with a third primer) is designed to anneal to the region upstream of the mutation site. Either 1–2 bases are added to the extension primer to produce two extension products from wild-type DNA and mutant DNA. MALDI-TOF mass spectrometry had a 99.1% accuracy with 98.9% sensitivity, 99.2% specificity [57,58].
(12) Digital PCR using microfluidic devices have the ability to perform highly parallel analysis in a single PCR step. Absolute quantification can be achieves rather than relative quantification compared to RT-PCR. Thus, point mutations, copy number variations, loss of heterozygosity or aneuploidy can be detected. Also, digital PCR can differentiate easily between high and low molecular weight DNA in a multiplex fashion. On the other hand, droplet Digital PCR is a modified method that removes the need for a reference standard curve as for qPCR. Furthermore, picodroplet dPCR facilitates simultaneous screening for multiple mutations from the same sample. On the other hand, multiplex digital PCR (dPCR) enables noninvasive and sensitive detection of circulating tumor DNA with performance unachievable by current molecular-detection approaches. This approach could potentially be adapted to the analysis of any locus amplified in CCFDNA [59–62].

## 8. Cancer Related DNA Methylation

Many tumor suppressor genes regulate cell cycle and promote apoptosis. The hypermethylation of the promoter of these genes results in the reduction or loss of gene expression. Methylation occurs as an addition of a methyl group to the number 5 carbon of the cytosine pyrimidine ring of DNA. In adult somatic tissues, DNA methylation typically occurs in a CpG dinucleotide segment which is regarded as one of the most significant epigenetic events. In particular, CpG methylation in the promoter region of certain genes has been observed as the earliest and most frequent alteration in some cancers by causing silencing of tumor suppressors. DNA methylation can be inherited or removed without changing the original DNA sequence [63–66]. A panel of epigenetic markers may possibly allow the detection of circulating tumor DNA in virtually all patients with different cancer types. Hence, the prognostic value of aberrant DNA methylation and therapeutic implications of demethylation of methylated genes could further improve the management of patients with different kinds of malignancies [65–67]. Examination of serum for circulating tumor DNA with abnormal methylation patterns offers a possible method for early detection of several cancers. Aberrant CpG island hypermethylation rarely occur in non-neoplastic and normally differentiated cells. Therefore, the DNA released from tumor cells can be detected with a notable degree of sensitivity, even in the presence of excess of DNA from normal cells and this represents a remarkable potential clinical application [68–70].

DNA methylation patterns measured in peripheral blood have great potential to be useful and informative biomarkers of cancer risk and prognosis. However, large systematic and unbiased prospective studies that consider biological plausibility and data analysis issues will be needed in order to develop a clinically feasible blood-based assay [68]. The central challenge is to validate DNA methylation as a cancer-specific biomarker assessed with reliable accuracy. Additionally, it is imperative to consider how such a screening mechanism can be implemented in populations at risk, especially in resource-poor settings [7,29,67–69].

The following genes (cell cycle, growth, differentiation & development) have been identified as frequently hypermethylated; hence, they are potential targets to be detected in CCFDNA.

*MLH1*: Colon, endometrial and gastric cancers;
*ER-beta* (Estrogen receptor beta) and *RAR-beta2*: Breast cancer;
*P16*: esophageal, lung, colon, liver and pancreas cancer;
*GSTP1* (Glutathione S-transferase P): hepatocellular carcinoma;
*14-3-3 sigma/stratifin*: breast cancers;
*BRCA1*: breast and ovarian cancers;
*VHL*: kidney tumors;
*RB*: retinoblastoma;
*TMEFF2*: Lung and various tumor types;
*DNA methyl transferase and MGMT*: various tumor types.

## 9. Microsatellite Alterations

### 9.1. Loss of Heterozygosity (LOH)

Loss of Heterozygosity (LOH) analysis of alleles at specific chromosomes of cell free plasma or serum DNA can add remarkable diagnostic and prognostic value for early evaluation of primary tumors such as mucosal melanoma, gastrointestinal stromal tumors, prostate carcinomas, and others. Studies have shown that LOH can indicate tumor recurrence and can correlate with tumor status as will be discussed in detail later on in this article as per tumor type [3,7,9]. From the methodological point of view it is very important to mention that circulating DNA is present in high and low molecular weight fractions, especially for breast and ovarian cancer. It has been demonstrated that LOH at different loci are found in the low molecular weight fraction. Thus, fractionation of circulating DNA is essential for achieving reliable results [7,9,11,12].

### 9.2. Microsatellite Instability (MSI)

Microsatellites are repeated sequences of DNA in which a short motif (usually 1–6 base pairs in length) is repeated 5–100 times. Expansions of microsatellite DNA repeats contribute to the inheritance of nearly 30 developmental and neurological disorders. Frequently, these disorders involve nearly all DNA transactions including replication, repair, recombination, and transcription [7,70,71]. MSI is the genomic evidence that results from malfunctioning of the Mismatch Repair System (MMR). DNA MMR corrects errors that spontaneously occur during DNA replication. Single base mismatches or short insertions and deletions are identified then subsequently excised and repaired. Cells with abnormally functioning MMR tend to accumulate errors rather than correcting those errors [71–73]. MSI can be detected in cell-free DNA and may increase the detection of cancer diagnosis and progression. MSI is associated with several cancer subtypes and testing for MSI-CCFDNA depends on a small number of known microsatellite loci or mismatch repair genes which represent some challenge to use this application [7,73].

## 10. Genetic Alterations in Cancer

Tumor location, morphology, differentiation, behavior and response to therapeutic modalities can be deduced from the tumor genomic signature. Variation in histology of the same type of tumors at the same organ (such as adenocarcinoma of breast) or different types of tumors in one organ (as in gastric carcinoma *vs.* stromal gastric tumor) can be explained by genetic variation between tumors [74–76]. One gene may express different alleles depending on the splicing events, and one protein could be produced by different genes such as human insulin. In general, variations of genomes between species or between members of the same species are attributed to genetic diversity as a result of new gene combinations (e.g., crossing over of chromosomes) and other genetic changes. In addition, comparative genomic studies have revealed notable genetic variation related to evolution between species.

Common fragile sites are hotspots for chromosome instability and co-localize with cancer-associated genomic rearrangements. One-third of the DNA methylation differences are not associated with any genetic variation and genomic instability may pre-exist in normal cells in the absence of exogenous replication stress [77–81]. Cancer is considered a systemic and multifactorial disease with the involvement of both genetic and environmental risk factors. Recent tumor genome sequencing confirmed that one tumor often consists of multiple cell subpopulations (clones) which bear different, but related, genetic profiles such as mutation and copy number variation (CNV) profiles. Identification of genetic variations (SNP, CNV) and epigenetic alterations from primary tumor cells has become a common method to discover genes critical to the development, progression, and therapeutic resistance of cancer [81–84].

Genetic polymorphisms in DNA repair genes may also influence individual variation in DNA repair capacity and may play an important role in carcinogenesis [9]. The genetic variation in genes associated with angiogenesis, major histocompatibility complexes (MHCs), the immune system, and inflammation may affect the final outcome and survival in cancer patients. In addition, the differences in cytochrome P450 (CYP) enzymes may play an important role in determining the efficacy of response to chemotherapy [83–85].

Genetic variants in growth factor signaling (e.g., *EGFR*, *ERBB2*, and *FGF1*) appear to also influence cancer risk. For example genetic variations have been associated with survival after diagnosis with breast cancer. Even in triple negative breast cancer there are novel variants as a result of splicing [85–89]. In gastric tumors, SNP variations in the *Fas* signaling pathway are consistent with associations of altered *Fas* signaling and/or apoptosis with risk of gastric carcinoma [90]. In hepatocellular carcinoma, the presence of A1762T/G1764A alteration was independently associated with the risk of HCC [91]. Genetic variations have been detected in most of the solid and hematopoietic tumors. These variations are currently being investigated to see whether they could be detected in CCFDNA.

## 11. Neoplasms

The median circulating plasma DNA concentration in patients with solid tumors is 17 ng/mL (range: 0.5–1600)—which was 3-fold higher than in healthy volunteers [92]. Higher CCFDNA concentrations were associated with worse overall survival. Most tumors have a variety of genetic changes and it will be very useful clinically if tumor-specific genomic aberrations/profile can be established. It appears that the target alleles or genes in CCFDNA molecules are the same as in primary tumors. There is high detection concordance for critical “hot-spot” mutations in matched CCFDNA and archival tumor tissue [9]. In summary, genetics alterations include decreased strand stability, presence of specific oncogenes, methylation or mutation of tumor suppressor genes, gene amplification, and microsatellite alterations.

### 11.1. Central Nervous System Tumors

Central nervous system tumors comprise approximately 120 histological subtypes and certain brain tumors. CNS tumors are associated with distinct profiles of circulating factors such as proteins, DNA fragments and miRNAs [93].

#### 11.1.1. Neuroblastoma

Detection of the *MYCN* in circulating DNA occurs in the early progression of *MYCN*-amplified neuroblastomas. This is strongly associated with rapid *tumor* progression and poor outcome, which is independent from stage of the tumor or the age of the patient. However, some *MYCN* non amplified (non-MNA) neuroblastomas show poor outcomes as well. For these cases, aberrant hypermethylation of the *DCR2* promoter of the serum DNA has been helpful in predicting prognosis, therapeutic efficacy, and detecting reoccurrence in (non MNA) neuroblastomas. Also, determination of the hypermethylation status of *RASSF1A* of serum DNA is another prognostic marker for the outcome in neuroblatoma patients [94–96].

#### 11.1.2. Gliomas

CCFDNA in glial tumors is useful for both LOH and aberrant gene promoter methylation. The levels are increased in high grade glioma patients. However, the sensitivity is moderate and specificity is high for both low- and high-grade oligodendrogliomas. The methylation status of the promoters for *p16/INK4a*, *MGMT*, *p73*, *RARβ* and LOH in chromosomes 1p, 19q, and 10q were detected in glioma tissue and plasma in most of the patients [97,98].

Glioblastoma grade 4 or multiform (GBM) is the most aggressive brain tumor in adults. GMB remains largely incurable despite multimodal intensive treatment regimens including surgical resection, radiation, and chemotherapy. *EGFRvIII* is a truncated extracellular mutant of the EGF receptor (*EGFR*) found in about a third of GBMs. It confers enhanced tumorigenic behavior and is associated with chemo- and radio-resistance. GBM patients testing positive for *EGFRvIII* in circulating DNA have a bleaker prognosis than those who do not. Targeting *EGFRvIII* positive tumors via vaccines or antibody-drug-conjugates represents a new challenging therapeutic avenue with potentially great clinical benefits. The circulating DNA status for *EGFRvIII* correlates with the analysis performed on the respective tumor samples, and its levels correlate with the extent of the tumor resection. Therefore, this may represent a strategy to screen patients for an anti-*EGFRvIII* therapy and to monitor the patients’ response to treatment [99]. Also, as in neuroblastoma, the patients with glial tumors are characterized by a higher frequency of *RASSF1A* hypermethylation that differentiates primary from metastatic brain cancers [100].

### 11.2. Breast Cancer

Circulating plasma DNA levels in breast cancer patients are significantly higher than in women with benign lesions and in control groups. In addition, circulating DNA levels are reduced after surgery. CCFDNA is associated with tumor size, tumor stage, tumor grade, lymph node involvement, *Her2/neu* and topoisomerase IIα expression [101–104]. Similar associations have been found with LOH of circulating DNA at the markers D3S1605, D10S1765, D12S1725, D13S218, and D17S855. The LOH at all markers was found in the fraction containing short DNA fragments than in the fraction containing the long DNA molecules, The most notable among these markers is LOH of D12S1725 which has been mapped to *cyclin D2* and is correlated with shorter overall survival [104,105]. Human epidermal growth factor receptor 2 (*HER2*) is amplified and overexpressed in 20%–25% of breast cancers. Amplified *HER2* in CCFDNA is a useful marker in patients with *HER2*-positive breast cancer. SNP/CNV analysis of circulating DNA reveals significant differences between patients with breast cancer and healthy controls during routine follow-up [106,107]. The detection of methylated genes in circulating DNA found in serum is also associated with the detection of circulating tumor cells in blood. DNA from serum correlates with progression and regional lymph nodes metastases while copy number of LINE1 (Long Interspersed Nuclear Element-1) from circulating DNA is correlated with tumor size [7,108–110].

Methylation status of *ERβ* (estrogen receptor β) and *RARβ2* (retinoic acid receptor β2) in serum could also potentially be used to predict invasive ductal breast carcinoma. Hence, concurrent *ERβ* and *RARβ2* methylation as well as loss of *ERβ* expression may serve as good prognostic markers [111]. The quantitative evaluation of *cyclin D2* and *RARβ2* methylation in CCFDNA provide valuable data for prediction. The methylation statuses of *CST6*, *APC*, and *RASSF1A* have been shown to be independent prognostic markers in breast cancer patients [62,111–113]. Methylated *RASSF1A*, *cyclin D2*, and *RARβ2* genes in CCFDNA are detected in 95% of breast cancer patients. In addition, aberrant hypermethylation of p16 and *CDH1* (E-cadherin or CD324 which is a tumor suppressor gene) are found in the plasma of 82% of breast cancer patients. Specifically, aberrant *p16* methylation in plasma and elevated serum CEA levels were associated with advanced tumor stage, tumor size, and extensive nodal metastasis as well [113–118].

Identification of biomarkers for monitoring the efficacy of neo-adjuvant chemotherapy in breast cancer patients is of the utmost importance in individualized therapy and reducing toxicity due to non-effective drugs. The methylation patterns in cell-free plasma DNA change after surgery, tamoxifen treatment, and after combined treatment [7,118–120]. Methylation of circulating tumor-specific DNA may also reflect changes in tumor burden in response to chemotherapy. *BRCA1* methylation frequency has been found to be different among responsive and non-responsive groups [121]. In addition, the kinetics of plasma DNA (ALU 115) is associated with response to neo-adjuvant chemotherapy in patients with locally confined breast cancer [118]. The mean DNA levels do not change significantly during chemotherapy. However, the integrity of serum DNA is higher in patients with increasing DNA levels and *vice versa*. During the course of adjuvant systemic chemotherapy, studies have identified that there are more longer DNA fragments that are released from non-apoptotic cells. These findings taken together might be helpful in evaluating the overall therapeutic response [121–123].

Other biomarkers of note include circulating *hTERT* (Telomerase Reverse Transcriptase in human). The levels were significantly different in the estrogen receptor (*ER*)(+)/progesterone receptor (*PgR*)(+) patients compared to the *ER*(−)/*PgR*(−) patients. Higher *hTERT* levels were also associated with higher human epidermal growth factor receptor (*HER*)-*2/Neu* expression. The levels of *hTERT* were significantly inversely correlated with the carbohydrate antigen (CA) 15.3 serum levels [111–113].

Another notable biomarker is vascular endothelial growth factor (*VEGF*). Among many endothelial regulators, *VEGF* is key in vasculogenesis and angiogenesis. Specifically, *VEGF* plays an important role in the growth and metastasis of breast cancer. Recent studies revealed that there is a significant negative correlation between *VEGF* and its soluble receptor *VEGFR1*, and a significant positive correlation between *VEGF* and cell-free serum DNA [123]. In summary, the circulating tumor DNA may provide the earliest measure of treatment response in patients. When assayed correctly, evaluation of CCFDNA in the context of breast cancer is informative, inherently specific, and highly sensitive as a biomarker for assessing the progression of breast cancer [124].

### 11.3. Female Gynecological System

#### 11.3.1. Endometrial Tumors

CCFDNA cannot be used as a significant screening tool for endometrial carcinoma (EC). However, the change in cell-free DNA may be a prognostic biomarker [125]. Plasma DNA integrity (longer DNA fragments) was found to be associated with endometrial carcinoma and high levels of CCFDNA were detected in patients with endometriosis. New molecular markers of endometrial cancer were found within anonymous DNA sequences located between microsatellite repeats 100 bp and 174 bp polymorphic fragments. These fragments appear to be homologous to a region within the *NFKB* and *DDR1* genes. Therefore, *NFKB1* and *DDR1* genes may be regarded as potential markers for some types of endometrial cancer. Also, the association between CCFDNA and *p53*-Ab might potentially serve as a marker in predicting prognosis and offers a possibility to individualize the treatment protocol [126–130]. One study showed that the female menstrual cycle does not significantly influence the CCFDNA serum level measurements and different time points of blood sampling in premenopausal women in this context would have negligible difference [129].

#### 11.3.2. Cervical Tumors

Plasma DNA levels are closely related with malignant transformation and development of cervical cancer. The serological detection of *MYOD1* promoter hypermethylation may be of potential use as a prognostic marker for discriminating cervical cancer patients at high risk for lymph node metastasis or relapse. Furthermore, unmethylated *CDH1*/*CDH13* in serum samples is most likely associated with better disease-free survival [62,131–133].

#### 11.3.3. Ovarian Tumors

Patients with epithelial ovarian cancer expressed higher amounts of CCFDNA and circulating cell-free mitochondrial DNA (CCFMDNA) in plasma compared with healthy controls. A significant difference between the epithelial ovarian cancer and endometriosis group was found in circulating cell-free mitochondrial DNA but not in circulating cell-free nuclear DNA [46]. Hypermethylation of *RASSF1A* (tumor suppressor gene) was found in circulating tumor-specific DNA in 43.1% of patients. There was no difference in the hypermethylation of the *RASSF1A* gene among various ovarian cancer subtypes. Hypermethylation of *RASSF1A* was more frequently encountered in stage III and IV than stage I and II tumors. In addition, *in vivo* experiments in mice showed that the levels of plasma DNA increase with the increased size of the ovarian tumor and decline after treatment. Also, the presence of *KRAS* mutations in mucinous ovarian cancer along with CCFDNA and *p53*-antibody in serous tumors was correlated with the highest risk of cancer progression [134–136].

It should be noted that CCFDNA may need to be fractionated into high- and low molecular-weight fractions (HMWF, LMWF) for LOH-profiling in some cases. It is necessary to do DNA-fractionation prior to analyzing circulating LOH. This methodology has allowed for the identification of LOH at D10S1765 and D6S1581 as novel blood-based biomarkers for ovarian cancer [137].

### 11.4. Hepatocellular Carcinoma (HCC)

High levels of circulating free plasma DNA (CFPDNA) have been detected in HCC and liver cirrhosis. Circulating DNA levels are closely correlated with tumor size and degree of differentiation. In addition, the levels of serum LINE-1 hypomethylation at initial presentation are correlated significantly with the presence of HBsAg, large tumor sizes, and advanced tumor stages. This is considered an independent prognostic factor of overall survival. However, circulating DNA has not been correlated with patient age, gender, levels of alpha-fetoprotein (AFP), or protein induced by vitamin K absence (PIVKA-II). In addition, it is still unclear whether the TNM stage correlates with CCFDNA or not within the context of HCC [138–140].

HCCs with high serum levels of CCFDNA also had increased levels of several inflammatory cytokine genes that may explain the inflammatory status in primary tumors with HCV-related HCC. DNA concentrations were significantly higher in HCC patients compared to HBV and HCV carriers without cancer, and to sero-negative individuals. Thus, CCFDNA may help guide monitoring the effect of viral infection in chronic liver disease and hepatic carcinoma as well [139–141].

Mutations of *p53* have also been reported as common mutations in solid tumors, including non-Hodgkins lymphoma, and have been implicated in drug resistance and poor prognosis. The mutation in *TP53* at codon 249 (Ser-249, considered a hallmark of mutagenesis by aflatoxin) and in *CTNNB1* (gene encoding beta-catenin) in CCFDNA may suggest a role of aflatoxin in hepatocarcinogenesis [142].

Hypermethylation of *RASSF1A* is considered an early event in the pathogenesis of HCC and can be found in premalignant liver tissues. Hypermethylation of *RASSF1A* sequences were detected in the sera of 93% of HCC patients, 58% of HBV carriers, and 8% of the healthy volunteers. Aberrant methylation of *p16* was detected in the plasma/serum samples of 81% of HCC. Patients with higher *RASSF1A* concentrations at diagnosis or one year after tumor resection showed poorer disease-free survival. Among HBV carriers who underwent HCC surveillance and subsequently developed HCC, the circulating concentration of *RASSF1A* increased significantly from the time of enrollment to cancer diagnosis [142–145]. LOH may also play an important role in hepatocarcinogenesis along with MSI. Specifically, microsatellite instability and loss of heterozygosity of D8S277, D8S298, and D8S1771 at chromosome 8p were detected on the plasma DNA of HCC patients [144–147].

### 11.5. Pancreatic Carcinoma

The current data suggests that, among patients with pancreatic disease, the methylation profiles of inflammatory disease and cancer are different. These data open a new venue for the development of biomarkers for differential diagnosis. Further investigation of diagnostic biomarkers for pancreatic cancer based on methylation in cell-free, circulating DNA appears to be warranted [148]. Pancreatic cancers are the fourth most common cause of cancer-related deaths in the United States. Unfortunately, effective screening programs for pancreatic cancer have not been developed, and patients are often detected incidentally or after development of symptoms. Assessment of DNA is an easy, simple, inexpensive screening method that could provide the drive to initiate more aggressive radiographic evaluation. For those who are fortunate to have early diagnosis, surgical resection offers the most promising therapy. However, despite adherence to rigorous surgical techniques and histopathology-based diagnosis, up to 80% of patients will suffer early, locoregional recurrences. Therefore, DNA can also be used as a postoperative surveillance tool [149,150]. Multiplexed array-mediated analysis of DNA methylation which included the promoters of *CCND2*, *SOCS1*, *THBS1*, *PLAU*, and *VHL* can detect ductal adenocarcinoma of the pancreas with significant accuracy in the early stage [150,151].

### 11.6. Gastrointestinal Tract

#### 11.6.1. Esophageal Tumors

There is significant difference in DNA methylation and integrity (short/long DNA fragments) between esophageal cancer patients and healthy control individuals. The plasma DNA concentrations and their integrity can serve as new diagnostic markers for screening and monitoring (pre *vs.* postoperative) patients with esophageal cancer. Quantification of circulating plasma DNA revealed that up to 61% of patients with esophageal carcinoma have detectable levels of methylated *DAPK* (Death-associated protein kinase) or *APC* (adenomatous polyposis coli gene) promoter DNA. The preoperative detection of *DAPK* and *APC* are significantly associated with unfavorable prognosis [152–154].

#### 11.6.2. Stomach Tumors

Plasma DNA concentration is higher in patients with gastric cancer compared with controls. Monitoring and early diagnosis of gastric cancer can be detected by epigenetic changes of cell-free serum DNA of *RUNX3*, *MGMT*, *p15*, and *hMLH1* hypermethylation using RTQ-PCR, fluorescence-based assay, and methylation-specific PCR (MSP). For postoperative monitoring, detection of methylation status of *CEA*, *P16*, *E-cadherin*, *RARbeta* and *CDH4* genes can be helpful as well [25,47,155,156].

#### 11.6.3. Colorectal Cancer (CRC)

Early detection of colorectal tumors through the identification of mutant DNA in serum or plasma is a clinically useful biomarker for screening, detecting, and monitoring therapy response. In addition, it could have a substantial impact on morbidity and mortality. Interestingly, tumor-derived (mutant) CCFDNA was found to be more fragmented than CCFDNA from normal tissues. More than half of the patients with early stage disease contain mutant DNA in their circulation. Other studies have shown that mutated circulating DNA may depend on tumor clonality, *i.e.*, whether the source is from tumor cells, tumor-associated stromal cells, or surrounding normal cells [157–163]. Methylation status of DNA in CRC has also been detected in colorectal tissues, stools, and peripheral blood. The aberrant methylation status of specific genes in the serum of patients with colorectal cancer has the potential to become a pre-therapeutic predictor of outcome. Examples of biomarkers include circulating methylated *SEPT9* (Septin) DNA. Methylated *SEPT9* DNA has been identified as a sensitive marker for screening and it is considered a valuable biomarker for the detection of minimally invasive colorectal cancer [54,164–166]. Other studies utilizing multivariate analysis have shown that methylated *HPP1* and/or *HLTF* serum DNA is independently associated with poor outcome and a relative risk of mortality. Interestingly, serum methylation of *hMLH1* was not associated with a higher risk of mortality [167–169].

*KRAS* mutation is a mediator of acquired resistance to *EGFR* blockade and these mutations can be detected in CCFDNA. *KRAS* mutations are frequent drivers of acquired resistance to cetuximab in colorectal cancers. Studies have suggested that the emergence of *KRAS* mutant clones can be detected non-invasively months before radiographic progression. Studies have also suggested that the early initiation of a *MEK* inhibitor may be a rational strategy for delaying or reversing drug resistance. Recently, point mutations of serum *KRAS2* have been identified which may provide information to substantially impact the management of late stage colorectal carcinoma (distant metastasis) [169–173]. Serum DNA integrity is significantly increased in CRC, even in cases of localized lesions and in advanced stage cancer. There are very high ALU-qPCR values with ALU115 primers, which lowered the serum DNA integrity, so the absolute serum DNA may be a better serum biomarker than DNA integrity. Therefore, a combined index of absolute concentration and integrity of serum DNA may decrease false negatives for cancer detection. On the other hand, ALU247 and ALU247/ALU115-qPCR biomarkers may be important in detecting and monitoring CRC patients in both early and late stages. In addition, the accuracy of early stage detection of tumor increased when CCFDNA was used in combination with CEA measurement [24,163,172–174]. Other mutations that have been identified as clinically significant include microsatellite instability, *BRAF*, and *SMAD4* [173,174]. Other genes with abnormal promoter methylation include *TMEFF2*, *NGFR*, and *p16* [45,174].

### 11.7. Head and Neck Tumors

#### 11.7.1. Nasopharyngeal Carcinoma

Concentrations of CCFDNA are significantly higher in nasopharyngeal carcinoma (NPC) patients than normal controls. The plasma DNA integrity index of the NPC patients was significantly higher than that of the healthy controls after radiotherapy. The integrity index of circulating DNA in NPC was calculated as the ratio of the two concentrations (201 bp/105 bp) and more intact circulating DNA would give a higher integrity index. Hence, the reduction in plasma DNA integrity index was observed in 70% NPC patients. Patients with persistent aberrations of plasma DNA integrity had significantly poorer survival probability than those with reduced DNA integrity after treatment [175,176]. The DNA integrity index in the plasma of the patients with head and neck squamous cell carcinoma (HNSCC) is increased in comparison with plasma from non-HNSCC control subjects. The lack of normalization of plasma DNA integrity index after surgical resection suggests the persistence of a population of cells with an altered pattern of DNA degradation despite the removal of the malignancy. No significant difference is noted between pre- and postoperative DNA integrity index in plasma samples from HNSCC patients [175–177].

Aberrant hypermethylated promoter DNA of at least one of the five following genes; *CDH1*, *p16*, *DAPK1*, *p15*, and *RASSF1A* was detectable in 71% of plasma of NPC patients before treatment. Hypermethylation of the promoter DNA of at least one in three genes (*CDH1*, *DAPK1,* and *p16*) was detectable in the post-treatment plasma of 38% of recurrent NPC patients and none of the patients in remission [176,178]. Despite the high response rate to chemotherapy in NPC, complete remission is uncommonly seen. Even though there is no significant difference in plasma DNA concentration of EBV-positive and -negative normal individuals, the EBV-DNA is a sensitive and specific marker in monitoring NPC by its ability to detect early recurrence. It also has an excellent correlation with treatment response [178].

#### 11.7.2. Thyroid Tumors

The detection of free circulating mutant *BRAF*/DNA in patients with papillary thyroid carcinoma (PTC) is possible and future studies are warranted to determine its clinical significance. An activating point mutation of the *BRAF* oncogene results in a V600E amino acid missense mutation that is found in a majority of PTCs [179].

### 11.8. Lymphoma, Leukemia

Quantification of plasma DNA may be useful for evaluating therapeutic effects and monitoring relapse in lymphoma/leukemia patients. Levels of CCFDNA in patients with Hodgkin lymphoma (HL), diffuse large B cell Lymphoma (DLBCL), and mantle cell lymphoma are significantly higher than in healthy individuals. Increased levels of plasma DNA are associated with advanced stage disease, presence of B-symptoms, elevated lactate dehydrogenase levels, and age >60 years. In DLBCL the *MGMT* promoter hypermethylation along with *p53* mutation are useful prognostic markers for favorable prognosis. In addition, rearranged immunoglobulin heavy chain DNA has been found in the plasma of patients with non-Hodgkin’s lymphoma and acute B cell leukemia [180–183].

### 11.9. Lung Cancer

#### 11.9.1. None Small Cell Lung Carcinoma (NSCLC)

Circulating plasma DNA levels are increased in lung cancer patients compared to normal healthy controls. The higher concentration has been associated with poor prognosis. Genetic variations due to methylation changes or LOH have been detected in the CCFDNA of NSCLCs. Complete or partial post treatment response to chemotherapy also correlates with no mutation detection compared to the pretreatment period. [18,36,184–187]. Evaluating methylation status of *14-3-3 sigma* of serum DNA in pretreatment condition and for P16M in pleural lavage are considered for survival. Hypermethylation of *RASSF1A*, *p14* (*ARF*) and *APC* are useful prognostic markers in patients receiving gemcitabine, and testing plasma DNA for *K-RAS* mutation is helpful in monitoring NSCLC patients receiving paclitaxel and carboplatin [187–196]. Furthermore, detection of epidermal growth factor receptor (*EGFR*) mutations using plasma DNA is essential to determine appropriate lung cancer treatment and monitoring [197].

#### 11.9.2. Small Cell Lung Carcinoma (SCLC)

In small cell carcinoma, the plasma is a more reliable source of tumor DNA than serum. Microsatellite markers or LOH are useful for the detection of alterations in the plasma DNA of SCLC patients [23,198].

#### 11.10. Male Genital Tract

##### Testicular Tumors

The CCFDNA levels are increased in testicular tumors and correlated with tumor stage, which includes cell-free serum mtDNA levels. Detecting levels of a 79-bp (mtDNA-79) and 220 bp (mtDNA-220) fragment of the mitochondrial specific 16S-RNA can be done by quantitative real-time PCR. Also, mtDNA-79 levels can be used when other conventional biomarkers (AFP, HCG, PAP, and LDH) are within normal ranges in testicular cancer patients. Hypermethylated status of CpG island of CCFDNA at *APC*, *GSTP1*, *PTGS2*, *p14* (*ARF*), *p16* (*INK*) and *RASSF1A* are useful in detecting and monitoring these tumors as well [199–201].

### 11.11. Urinary System

Tumor DNA derived from renal cell carcinoma, bladder cancer, or prostate cancer is considerably detectable in more than 50% of plasma/serum samples and in more than 70% of urine samples from these patients [202].

#### 11.11.1. Kidney Tumors

Surgical resection of kidney cancer is an effective therapy if detected at early stage. Renal cancer of all types can potentially be diagnosed by detecting promoter hypermethylation with a panel of genes including *VHL*, *p16/CDKN2a*, *p14ARF*, *APC*, *RASSF1A*, and *Timp-3*. Detection can be achieved with 88% sensitivity and almost 100% specificity since hypermethylation is not observed in normal and benign disease controls [202,203].

#### 11.11.2. Prostate Carcinoma (PCa)

Circulating DNA was detected in prostate cancer patients compared to normal individuals and men with benign prostate hypertrophy (BPH). It is also correlated with circulating tumor cells, tumor stage, and Gleason score. One reason for a high CCFDNA concentration is decreased DNase activity. LOH and genetic aberrations such as allelic imbalance (AI) and epigenetic changes of promoter hypermethylation (methylation of *RASSF1*, *RARB2*, and *GSTP1*) have also been detected in CCFDNA of prostate cancer patients [204–209].

Studies to date have shown significant associations between LOH and increasing Gleason scores for the marker combinations of D6S1631, D8S286, D9S171, D8S286 and D9S171 [207–211]. The methylation of the *GSTP1* gene was found in 25% of free plasma DNA and in 94% of tissue samples. *GSTP1* gene methylation is also associated with increased risk of PCA despite negative prostate biopsy. However, studies evaluating the combination of CCFDNA and PSA assay gave 89% sensitivity in detecting PCa. The combination of DNA load and promoter methylation status identified 88% of PCa. Circulating mtDNA levels did not distinguish between patients with prostate cancer and BPH. However, there was a significant increase in short mtDNA fragments in patients with early PSA increase after radical prostatectomy. Additional studies have shown that *GSTP1* hypermethylation was only present in a small percentage of circulating DNA, and concentrations of apoptotic *PTGS2* fragments discriminate sensitivity (88%) and specificity (64%) between BPH and PCa. The apoptotic index (AI) was more specific (82%) but less sensitive (70%) [212–216].

The principal source of CCFDNA in prostate carcinoma is due to cell death during treatment. This feature could correspond well with tumor activity. Thus, CCFDNA can be an important noninvasive and useful biomarker especially for follow up in patients with prostate cancer [215–218].

### 11.12. Skin

#### 11.12.1. Malignant Melanoma

Several biomarkers detectable through the valuation of circulating DNA can be utilized within the context of melanoma. For example, hypermethylated *ER-alpha* is a significant factor in melanoma progression and is associated with unfavorable prognosis. *BRAF*V600E is the most represented somatic point mutation in cutaneous melanoma. Quantitative analysis has also shown higher levels of CCFDNA in patients compared to controls. These features are helpful in monitoring the disease in late stage (stage IV) melanoma but are unsatisfactory for the early detection of melanoma [219,220].

LOH at microsatellite markers D1S243, D6S311, D9S161 and D19S246 in the plasma is also associated with malignant mucosal melanoma (MMM). These loci are suitable for identifying cancer related DNA of MMM. Hence, the analysis of LOH in circulating plasma DNA is useful marker for diagnosis of recurrence and metastasis MMM [220–222]. Also, detection of *TFPI2*-methylated DNA in the serum of patients with resected melanoma is a sensitive and specific biomarker of recurrence or metastatic melanoma as well [222].

#### 11.12.2. Squamous Cell Carcinoma (SCC)

In 90% of squamous cell carcinomas of the oral cavity, there is a microsatellite alteration in serum DNA that is identical to those in the corresponding tumor DNA. This may provide valuable prognostic information and serve as a guide for future therapy. There are nine microsatellite loci of LOH that have been identified on chromosomes 2, 3, and 21 related to the SCC of oral cavity [223].

## 12. Conclusions

Circulating cell-free DNA (CCFDNA) has been suggested as a cancer biomarker. Several methods have been implemented to determine the quantitative and qualitative tumor-specific alterations of (CCFDNA), such as DNA strand integrity, gene amplification, gene mutations, gene methylation and microsatellite abnormalities as diagnostic, prognostic, and monitoring markers in cancer patients. Significant progress has been accomplished so far and still more work needs to be done to optimize the best clinical use of this application.
